# TERT Promoter Mutations and Telomerase in Melanoma

**DOI:** 10.1155/2022/6300329

**Published:** 2022-07-19

**Authors:** Yuchen Guo, Yi Chen, Lianghui Zhang, Ling Ma, Keyu Jiang, Gang Yao, Lingjun Zhu

**Affiliations:** ^1^Department of Oncology, The First Affiliated Hospital of Nanjing Medical University, Nanjing, China; ^2^Department of Plastic and Burns Surgery, The First Affiliated Hospital of Nanjing Medical University, Nanjing, China; ^3^Department of Oncology, Sir Run Run Hospital, Nanjing Medical University, Nanjing, China

## Abstract

Malignant melanoma is an extremely malignant tumor with a high mortality rate and an increasing incidence with a high mutation load. The frequency of mutations in the TERT promoter exceeds the frequency of any known noncoding mutations in melanoma. A growing number of recent studies suggest that the most common mutations in the TERT promoter (ATG start site −124C>T and −146C>T) are associated with increased TERT mRNA expression, telomerase activity, telomere length, and poor prognosis. Recently, it has been shown that TERT promoter mutations are more correlated with the occurrence, development, invasion, and metastasis of melanoma, as well as emerging approaches such as the therapeutic potential of chemical inhibition of TERT promoter mutations, direct telomerase inhibitors, combined targeted therapy, and immunotherapies. In this review, we describe the latest advances in the role of TERT promoter mutations and telomerase in promoting the occurrence, development, and poor prognosis of melanoma and discuss the clinical significance of the TERT promoter and telomerase in the treatment of melanoma.

## 1. Introduction

**Figure 1:**
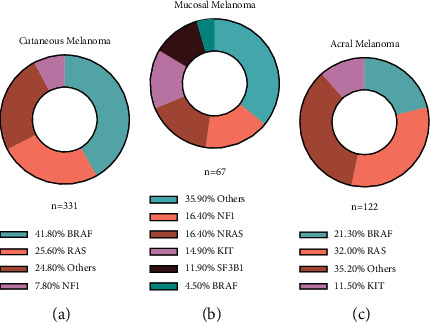
The percentage of the major mutations in melanoma. (a) Cutaneous melanoma. (b) Mucosal melanoma. (c) Acral melanoma.

Malignant melanoma is one of the deadliest forms of skin cancer, and its incidence is increasing year by year [1]. Due to the high rates of recurrence and metastasis, the overall prognosis of melanoma is awful and dismal, especially when diagnosed at advanced stages. Indeed, the 5-year survival rate for metastatic melanoma is less than 41.6%. Studies have shown that conventional chemotherapy and radiation have limited effects on melanoma, which may be associated with the production of melanin by cancer cells [2]. The mutation load of the melanoma genome is the highest of all types of cancers; we have summarized the percentage of the major mutations in melanoma based on the previously published studies of whole-genome sequencing of melanoma [3–5] (Figure 1). Besides, current therapeutic strategies led by targeted therapy and novel immunotherapy offer new hope for the treatment of melanoma [6].

Recently, a growing number of studies have been focusing on telomerase regulation and telomere shortening for their important roles in tumor inhibition. Telomerase is a ribonuclease consisting of RNA template (TERC) and reverse transcriptase catalytic subunit (TERT) [7]. Telomerase synthesizes new telomere DNA to counteract the telomere shortening, which normally occurs during DNA replication. It plays an important role in maintaining telomere stability, genome integrity, long-term cell activity, and potential for continued proliferation, and its activity is regulated by the TERT gene [8]. Telomerase activity in normal human tissues is inhibited, and active telomerase can only be detected in cells that must continue to divide, such as hematopoietic cells, stem cells, and germ cells. Besides, increased telomerase activity can also be detected in tumor cells, which participate in malignant transformation [9].

The hTERT gene is 42 kb long and located on chromosome 5 with 16 exons. TERT promoter mutations can occur early in the tumor and are considered to be one of the major mutations in tumors such as melanoma, glioblastoma, and hepatocellular carcinoma. In melanoma, TERT promoter mutations are the most common mutations in noncoding regulatory regions [10]. The frequency of mutations in the TERT promoter exceeds the frequency of any known noncoding gene mutations in melanoma [11], and the most common mutations in the TERT promoter (ATG start site −124C>T and −146C>T) are associated with increased TERT mRNA expression, telomerase activity, telomere length and poor prognosis [12].

This review has described the research progress of TERT promoter mutations and telomerase in melanoma, the molecular mechanism of TERT promoter mutations, and the future treatment of melanoma patients with TERT promoter mutations.

## 2. Molecular Mechanism of TERT Promoter Mutation

In recent years, many studies have confirmed the relationship between TERT promoter mutations and maintenance of telomerase activity, as well as the occurrence, progression, and prognosis of cancer [13–19]. TERT promoter mutations have become a key mechanism for the continuous upregulation of human telomerase to maintain unlimited growth of telomere. TERT promoter mutations are common mutations in melanoma and were first described in familial and sporadic melanoma [20]. A total of seven TERT promoter mutations were identified in melanoma, −124C>T, −146C>T, −124/125CC>TT, −138/139CC>TT, −136C>T, −100C>T, and family variants −57A>C [21]. The ETS (E26 transformation specific) family transcription factor is the key regulator for malignant tumor cell proliferation and invasion by mediating the transcription of the invasion-related pate factors [22]. These TERT promoter mutations have been shown to establish ETS transcription factor binding sites in the promoter, upregulate TERT transcription activity and expression, and upregulate telomerase activity. Thereby it can maintain telomerase length and eliminate the telomere senescence barrier, which gives cancer cells unlimited proliferation potential to promote cell immortalization and lead to the growth of melanoma. In contrast, in human cancer cell lines, knockdown of TERT causes a rapid decline in cell proliferation and growth [13].

The wild-type (WT) TERT promoter is enriched with repressive histone marks such as H3K27me3, which is associated with transcriptional silencing of the TERT gene [23]. TERT promoter mutations will increase TERT transcription and expression by creating new binding sites for transcription factors of the ETS family [20]. Li and Tergaonkar identified that a proximal ETS binding motif is created next to a p52 half-site which facilitates cooperative binding of ETS1 and p52. At the same time, the critical residues required for dimerization of ETS and p52 are absent in both WT and C228T TERT promoters. The binding partner p52 of ETS factor-ETS1 in cells containing C250T TERT mutation drives TERT transcription [23, 24]. In addition, Li et al. found that the noncanonical NF*κ*B signal is necessary to drive the TERT transcription, especially in the C250T mutant TERT promoter, which directly drives transcription by interacting with ETS factors. Consistent with their biochemical data, knockdown of p52 during atypical NF*κ*B activation eliminated tumorigenesis in a mouse model of C250T mutant glioblastoma cell transplantation. The C250T mutation produces a half-site NF*κ*B consensus sequence (5′-GGGGG-3′ or 5′-GGAA-3′), and compared to WT TERT, increased p52 binding was observed in the mutant promoter. Their study further proved that p52 binds to a new half-site in cells with C250T but not C228T mutations, and the expression of TERT has increased [24]. What is more, Li and Tergaonkar further discovered that C228T mutations will lead to an epigenetic switch that occurs resulting in the association of active H3K4me2/3 marks and GABP recruitment on the mutant allele [23]. Stabilization of GABP, an ETS family transcription factor, on mutant TERT promoter leads to TERT reactivation [23].

**Figure 2:**
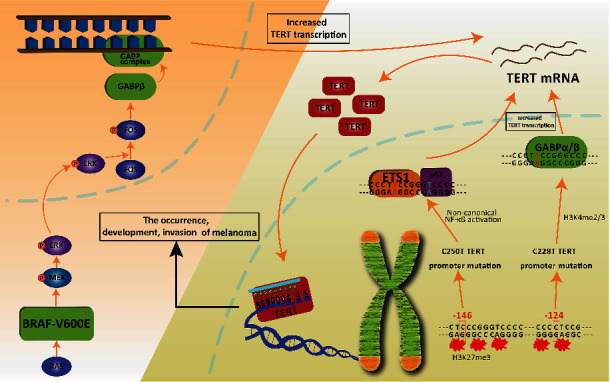
The mechanisms of TERT promoter mutations in occurrence, development, invasion, and therapeutic potentials in melanoma. The wild-type TERT promoter is enriched with repressive histone marks such as H3K27me3. In the context of C250T TERT promoter mutation, a proximal ETS binding motif is created next to a p52 half-site, which facilitates cooperative binding of ETS1 and p52. Next, during deregulated noncanonical NF*κ*B signaling in C250T mutant cancers, stabilization of ETS-p52 dimer on mutant TERT promoter results in elevated TERT expression. The critical residues (in blue) required for dimerization of ETS and p52 are absent in both WT and C228T TERT promoters. C228T mutation will lead to an epigenetic switch that occurs resulting in the association of active H3K4me2/3 marks and GABP recruitment on the mutant allele. Stabilization of GABP on mutant TERT promoter leads to TERT reactivation. Besides, BRAF V600E/MAPK pathway phosphorylates and activates FOS, which in turn acts as a transcription factor to bind and activate the GABPB promoter, increasing the expression of GABPB and driving the formation of the GABPA-GABPB complex. The formation of the GABP complex selectively binds and activates the mutant TERT promoter, upregulating TERT expression. Next, TERT promoter mutations will increase TERT transcription activity. Telomerase activity mainly depends on the amount of TERT. Increased telomerase activity will maintain telomere length and promote cell immortalization and melanoma growth.

In addition, the coexistence of TERT promoter mutations and BRAF V600E is thought to be a strong genetic background for promoting the aggressiveness of human cancers. Liu et al. proved that the BRAF V600E/MAPK pathway phosphorylates and activates FOS, which in turn acts as a transcription factor to bind and activate the GABPB promoter, increasing the expression of GABP*β* and driving the formation of the GABP*α*-GABP*β* complex. The complex selectively binds and activates the mutant TERT promoter, upregulating TERT expression [25, 26]. Based on the recent work by several independent groups, we summarize a mechanism diagram (Figure 2).

All in all, TERT promoter mutations will increase TERT transcription activity and TERT mRNA expression. Telomerase reactivation mostly depends on the amount of TERT in cells [27]. Thus, after the above series of changes, increased telomerase activity has been observed so that cells will proliferate indefinitely and melanoma will grow rapidly.

## 3. Multifunction of TERT Promoter Mutations in Malignant Melanoma

### 3.1. Roles in Melanoma Occurrence and Development

Telomerase is silent in most differentiated human cells, mainly due to the transcriptional inhibition of the TERT gene, the catalytic component of telomerase [27]. However, cancer cells can bypass this limitation by reactivating telomerase. Telomerase activity mainly depends on the amount of TERT [27]. Chiba et al. found that cells with TERT promoter mutations carry abnormally long telomeres, proved the causal relationship between TERT promoter mutations and telomere maintenance, and showed that TERT promoter mutations can upregulate TERT levels and telomerase activity, which is enough to inhibit telomere erosion [14].

TERT promoter mutations in malignant melanoma mainly occur on two hot spots on chromosome 5, namely, C228T mutation and C250T mutation [20], which correspond to −124C>T and −146C>T in the translation initiation site, respectively [11, 28]. Based on the results of RT-qPCR, Lee et al. reported that the hot spots of TERT promoter −124C>T, −146C>T mutation, and −138/139CC>TT mutation were all related to TERT overexpression in melanoma and suggested that −146C>T and −138/139CC>TT mutations could biologically contribute to melanoma [29]. The relationship between increased TERT gene expression and −124/125CC>TT and −146C>T mutations was also confirmed by the experiment of Shaughnessy et al. [21]. Research by Seungjae Lee et al. showed that −124C>T genotype is more effective than −146C>T genotype in promoting TERT expression [29], and −146C>T and −124C>T mutations increase telomerase activity [29]. In addition to somatic mutations mentioned above, mutations of the germline from −57A>C found in familial melanoma have shown similar effects [20]. Besides, TERT promoter mutations elevate TERT expression by modulating the transcriptional activity. Horn et al. reported that TERT promoter mutations increased TERT transcription by 1.5–2 times when tested in melanoma tumor cells [20]. In another study, Franklin et al. described two independent mutations C228T and C250T in the core promoter of TERT, which resulted in a new common binding motif for the E-26 (ETS) transcription factor. These TERT promoter mutations increased the transcriptional activity of the TERT promoter by 2–4 times, resulting in elevated TERT gene expression and telomerase levels in cancers [28]. Furthermore, the research conducted by Heidenreich et al. showed indirect evidence that all cancer-related TERT promoter mutations could upregulate TERT expression in melanoma [11]. The study of Barthel et al. about telomere length of 31 cancer types from the Cancer Genome Atlas (TCGA) cohort and the study of Arita et al. showed that regardless of the TERT promoter methylation status, TERT expression was increased in almost all tumors with mutations in the TERT promoter [30], which in turn affected telomerase activity in tumors [10].

Studies have shown that although telomerase expression is increased in tumor cells with TERT promoter mutations, the observed telomeres are still very short [31], suggesting that the key effect of TERT promoter may occur after telomeres become extremely short [32]. Chiba et al. demonstrated that, in the initial stage of melanoma, TERT promoter mutations did not prevent the overall shortening of telomeres but prolonged cell life by repairing the shortest telomeres. In the next stage, critically short telomeres lead to genome instability and further upregulated telomerase to maintain cell proliferation [32].

Telomerase activation is known to be a key step in immortalizing more than 90% of human tumors [33]. Increased TERT expression and telomerase activation caused by TERT promoter mutations promote tumor cell growth by stabilizing telomere length, which eliminates the telomere aging barrier and gives cancer cells unlimited proliferation potential necessary for immortalization and malignant transformation [15]. In addition to genetic variation, the risk of melanoma is positively associated with telomere length in the entire population [34]. A study based on 1469 melanoma patients and 1158 healthy controls showed a statistical association between increased telomere length and melanoma risk, revealing that telomeres carrying TERT promoter mutations were longer than noncarriers [16]. Recently, a single nucleotide polymorphism rs2853669 variant (at position −245) has been involved in the regulation of TERT promoter mutations on melanoma survival and recurrence [35]. Shaughnessy et al. further verified that TERT promoter mutations modulated melanoma survival and recurrence by targeting telomere length [21]. Chiba et al. used CRISPR technology to generate C228T mutations in the TERT promoter region in human pluripotent stem cells and found that these cells still constitutively expressed TERT and telomerase after terminal differentiation, while wild-type (WT) stem cells stopped TERT transcription after cell differentiation. Compared with normal cells, differentiated cells with TERT promoter mutations carried longer telomeres and thus eliminated the replication aging caused by telomere wear [14]. When stem cells differentiate into somatic cells, which can normally silence telomerase, cells with TERT promoter mutations cannot silence TERT expression, resulting in increased telomerase activity and abnormally long telomeres. As a result, the risk of cutaneous melanoma metastasis increases [36]. Therefore, TERT promoter mutations are sufficient to overcome the proliferation barrier caused by telomere shortening. These data prove that TERT promoter mutations can promote the immortalization of neoplastic cancer cells and tumorigenesis [14].

What is more, increased telomerase activity will promote the rapid growth of melanoma. Analysis of the experimental data of Nagore et al. showed that the frequency of TERT promoter mutations in fast-growing melanoma was almost twice as higher as that in slow-growing melanoma. In this experiment, after adjusting for age, location, histological subtype, Breslow thickness, ulcer, and tumor mitosis rate, TERT promoter mutations were still associated with fast-growing melanoma [17]. In the melanoma mouse model, inhibition of telomerase activity significantly reduced the tumor invasion and metastasis potential [18], indicating the relationship between telomerase activity and melanoma growth.

### 3.2. Roles in Melanoma Invasion and Metastasis

Melanoma is a malignant tumor with a very low survival rate. It has a poor prognosis and is prone to metastasis [6]. Malignant melanoma can spread through the blood to distant organs (hematogenous metastasis) or through the lymphatic system to locoregional skin and lymph nodes (lymphatic metastasis) [19]. Melanocytic Spitzoid lesions include benign Spitz nevus, atypical Spitz tumor (AST), and Spitzoid melanoma. In the study of Lee et al., TERT promoter mutations were found in Spitzoid melanoma patients with blood metastasis, but none of the TERT promoter mutations were found in patients with good outcomes [37]. The results showed that the presence of TERT promoter mutations was significantly related to the risk of extra lymph node metastasis or death from Spitzoid melanoma. TERT promoter mutations were one of the most important predictors of hematogenous metastasis and served as a predictive index of aggressive clinical behavior [37]. TERT promoter mutations can stratify clinical risk for patients with Spitzoid melanoma, but the application of TERT promoter mutations detection for risk stratification in clinical practice still needs large-scale verification [37]. The increased frequency of TERT promoter mutations in metastatic melanoma is associated with higher invasiveness [38]. The retrospective cohort study conducted by Rees et al. has unraveled that TERT promoter mutations are risk factors for hematogenous metastasis, supporting the idea that adjuvant targeted therapy can help prevent patients carrying this mutation from spreading the disease through blood [19]. Furthermore, TERT promoter mutations are associated with visceral spreading in melanoma of the trunk, which may explain that trunk melanoma can skip local metastases by promoting visceral transmission [39].

In the experimental cohort of Ekedahl et al., the mutation rate of TERT promoter in the tumor tissue of patients with nonacral cutaneous metastatic melanoma was high, which may represent a greater metastatic potential in the primary tumor of TERT promoter mutations [40]. Potentially, in addition to the effects on telomere length and proliferation rate, telomerase may also enhance transfer potential through other mechanisms [40], and higher telomerase activity is also associated with higher tumor proliferation rate and early metastasis [41]. In contrast, inhibiting the activity of telomerase in melanoma cell lines induces cell differentiation and reduces its ability to invade and metastasize [42].

### 3.3. TERT Promoter Mutations Are Involved in Tumor Immune Microenvironment

TERT promoter mutations increase TERT transcription activity, TERT expression, and telomerase activity. TERT is a self-antigen constitutively expressed in a variety of tumors, so it is an important target for anticancer immunotherapy. TERT mutations are significantly associated with higher TMB values, neoantigen load, and immunosuppressive microenvironment [43]. Although previous studies have not been as good as expected, TERT can still provide personalized immunotherapy when combined with immune checkpoint suppression. As an intracellular protein, TERT can only be recognized by T cells as short peptides containing 8–16 amino acids, processed within the cell and presented within the cell. Preliminary experiments about TERT immunology focused on the binding of TERT peptides to the MHC class I major histocompatibility complex (MHC I) molecule. MHC class I (MHC I) molecules are expressed by almost all cell types. They present the target antigen peptides to induce CD8+ Cytotoxic T lymphocyte (CTL) expressing complementary T-cell receptor (TCR). Therefore, the significant question is whether endogenous TERT can be processed and presented in the context of MHC I and become a target of CD8+ T lymphocytes, thus activating T-cell toxicity [44]. Besides, cancer cells can present TERT peptides via CD4+ or CD8+ T cells. In vitro, TERT protein is highly immunogenic to peripheral blood T lymphocytes in healthy people and cancer patients, indicating that TERT-reactive T-cell precursors are present in the blood and are not missing in the thymus. This finding is important because immunization does not result in the regeneration of antigen-specific T cells but selectively expands the preexisting reactive clones in the T-cell bank [45].

In addition, NF*κ*B is a key regulator of innate immunity and adaptive immunity and is essential for host defense [46]. Activated NF*κ*B regulates the expression of target genes, which are responsible for cell survival, proliferation, differentiation, and establishing an appropriate immune response for host defense [47]. NF*κ*B has also been reported to be related to tumorigenesis and chemotherapy resistance [47]. In addition, Li et al. found that the noncanonical NF*κ*B signal was necessary to drive the TERT transcription, especially in the C250T mutant TERT promoter, which directly drove transcription by interacting with ETS factors. Besides, ETS1/2 heterodimerized with p52 in the C250T region and synergistically activated the expression of the TERT gene, thus proving the atypical role of NF*κ*B in the activation of telomerase in cancer cells with TERT promoter mutations [24].

## 4. Potential Clinical Application of TERT Promoter Mutations

### 4.1. Effect of TERT Promoter Mutations on the Diagnosis and Prognosis of Melanoma

#### 4.1.1. TERT Promoter Mutations and Diagnosis of Melanoma

Among several related tumor types, TERT promoter mutations seem to constitute a new prognostic biomarker, with potential application prospects in presurgery diagnosis and patient follow-up [48]. TERT promoter mutations are prevalent in malignant melanoma but rarely in melanocyte nevus [49]. A retrospective study by Walton et al. also concluded that hot TERT promoter mutations are more common in recurrent melanoma than recurrent nevus, and TERT promoter mutations could serve as a diagnostic clue in histologically ambiguous cases [50]. Thus, hot spot TERT promoter mutations may help distinguish melanoma from nevus [49].

Genetic testing for targetable somatic mutations is considered mandatory by the European Guidelines in the context of diagnosis, treatment, and follow-up of cutaneous melanomas in patients with advanced disease (unresectable stage III or stage IV) and is highly recommended in high-risk resected disease (stage IIC or stages IIIB-IIIC) [51]. Simona et al.'s findings encouraged the analysis of TERT mutations in melanomas that originate in the trunk because they are more likely to progress to the internal organs. TERT screening will help select patients who may benefit from more intensive follow-up protocols and start treatment quickly [39, 52].

#### 4.1.2. TERT Promoter Mutations and Prognosis of Melanoma

TERT promoter mutations are biomarkers of poor melanoma outcomes, and mutations leading to increased TERT expression may play a role in tumor growth. In previous reports, TERT promoter mutations have been linked to poor survival of melanoma patients [38]. Previous studies have described the link between increased telomerase activity and poor prognosis in melanoma, including ulcers, vascular invasion, high mitotic rate, and increased Breslow thickness [53]. In primary melanoma with TERT promoter mutations, there is a tendency for the tumor thickness to increase [27]. In addition, higher telomerase activity is also associated with a higher tumor proliferation rate and early metastasis [41]. In contrast, inhibiting the activity of telomerase in melanoma cell lines induces cell differentiation and reduces its ability to invade and metastasize [42]. Besides, some studies have shown that the longer the telomere length, the greater the risk of cutaneous melanoma development [54]. In addition, the positive expression of TERT in primary melanoma is related to the reduced survival rate of single-factor analysis; and in metastatic melanoma, there is also a trend between the positive expression of TERT and the decreased survival rate [27].

TERT promoter mutations also have a certain effect on the survival of melanoma patients. Hugdahl et al. found that although there are many sites for TERT promoter mutations in melanoma, there is no difference in survival rates among patients with different TERT promoter mutations [27]. However, Juan et al. found that −138/−139 CC>TT tandem mutation is associated with the worst disease-free survival and melanoma-specific survival, worse than −124C>T and −146C>T mutation [55]. Combined with previous research, it is speculated that tandem mutations may cause greater genomic instability than common TERT promoter mutations and therefore are associated with the worst survival rate of melanoma [55]. Also, melanoma patients with TERT promoter mutations have shorter disease-free survival than patients without such mutations [35]. Compared with patients with −124C>T mutations, patients with −146C>T mutations showed significantly worse progression-free survival (PFS) and a twofold increase in the risk of progression. This trend has also been observed for overall survival. Patients with −126C>T mutations have a worse prognosis than melanoma patients with −124C>T mutations [56]. However, further independent prospective studies are needed to assess the reliability of TERT promoter mutations as an independent prognostic factor for melanoma [57].

### 4.2. The Future of Therapeutic Potentials of TERT Promoter Mutations and Telomerase

TERT promoter mutations are unique to tumor cells and do not exist in surrounding normal tissues; thus, any intervention that specifically targets its mode of operation may affect the survival of tumor cells [14]. According to the existing research about TERT promoter mutations and telomere, the prominent role of telomerase in human tumors promotes the development of telomerase inhibitors to inhibit tumor growth. Besides, gene therapy and immunotherapy are considered to be possible to control TERT expression in tumors [58].

#### 4.2.1. Gene Editing

Li et al. used single-guide RNA (sgRNA) to guide and catalyze the damaged *C. jejuni* CRISPR-related protein 9 fusion adenine base editor (CjABE) to correct the −124C>T TERT promoter mutation to −124C. The modification prevents the members of the ETS transcription factor family from binding to the TERT promoter and reduces TERT transcription and TERT protein expression. It also induces the senescence of cancer cells and inhibits the proliferation of cancer cells. At the same time, local injection of adenoassociated virus expressing CjABE guided by sgRNA can inhibit the growth of glioma with TERT promoter mutations. These studies validate the feasibility of gene editing as a cancer treatment, and the activated TERT promoter mutation is a cancer-specific therapeutic target [59].

#### 4.2.2. Targeting Transcription Factors

TERT promoter mutations associated with cancer formation have created new binding sites for ETS (E26) family transcription factors and increased TERT expression. GABP (GA binding-protein) is an ETS transcription factor [60]; the GABP transcription factor is a polymer composed of a DNA-bound GABP*α* subunit and a transactivated GABP*β* subunit [61]. The study by Mancini et al. confirmed that GABP*β*1L can be used as a potential target for the treatment of tumor cells with TERT promoter mutations. By destroying the expression of GABP*β*1L, TERT expression was reduced, which ultimately led to the loss of telomeres and cell death in TERT promoter mutant cells. Reduction of GABP*β*1L in orthotopic xenotransplanted mice with TERT promoter mutant glioblastoma cells reduced the tumor burden and prolonged the overall survival time of mice [62]. Therefore, by inhibiting the expression of transcription factors, it is hoped that the tumor burden of patients with TERT promoter mutations can be reduced and the survival time will be prolonged.

#### 4.2.3. Targeting Telomerase

Telomerase has a long history as a cancer target, but only one direct telomerase inhibitor imetelstat has entered clinical trials. Imetelstat is a lipidated 13mer thiophosphoramidic acid oligonucleotide complementary to the TERC template region, which can competitively inhibit telomerase activity, cancer cell viability, and tumor growth in vitro and mouse xenograft models. Imetelstat can promote progressive telomere wear, which leads to activation of the DNA damage response and cell death after a prolonged delay period [63].

In a model of melanoma mouse, inhibition of telomerase activity can significantly reduce the tumor's potential for invasion and metastatic [18]. Therefore, telomerase inhibition may be a future intervention for melanoma with TERT promoter mutations, and more experiments are needed to further explore and verify. The results of an early clinical trial of telomerase inhibitors showed that the median progression-free survival and overall survival of patients with shorter telomeres compared with patients with longer or intermediate telomeres tend to improve [64]. Although oligonucleotides and immunotherapeutics targeting telomerase are progressing fastest in clinical development, small molecule inhibitors (such as BIBR1532) have produced promising preclinical results. BIBR1532 is a noncompetitive small molecule inhibitor of telomerase, which can mediate progressive telomere shortening and prolonged replication senescence after treatment in cancer cells [65]. Structural analysis using *Tribolium castaneum* TERT showed that BIBR1532 disrupted telomerase assembly (CR4/5) by binding to the conserved hydrophobic pocket (FVYL motif) of TERT and disrupting the interaction with the TERC activation domain [65]. Therefore, telomerase inhibition still is an effective intervention for cells with TERT promoter mutations [66]. Considering the heterogeneity of tumors and the ability of most cancer cells to quickly adapt to pharmacological challenges, successful strategies targeting telomerase may need to be combined with targeted therapy or immunotherapy to achieve the best antitumor effect [63].

#### 4.2.4. Combined Targeted Therapy

TERT promoter mutations lead to increased TERT expression and telomerase activity and are common in BRAF V600 mutant melanoma [56]. According to the study by Bianco et al., they evaluated the impact of the two most common TERT promoter mutations on the prognosis of melanoma patients treated with MAPK Inhibitor. The results showed that the −146C>T mutation had a twofold increase in the risk of progression compared with the −124C>T mutation, indicating that the two TERT promoter mutations have different roles in MAPK pathway blockade. Although, as mentioned above, it is possible that the NF*κ*B signaling pathway does not work in cell lines with −146C>T mutations, MAPK blockade cannot be overcome. However, in melanoma cell lines, both TERT transcription and telomerase activity decreased sharply after short-term exposure to MAPK inhibitors, regardless of the TERT promoter mutations [56]. Besides, Tan et al. used models of thyroid cancer, melanoma, and colon cancer cells. They found that Dabrafenib and Trametinib induced strong apoptosis in cancer cells carrying both BRAF V600E and TERT promoter mutations but had a little proapoptotic effect in cells that only carry BRAF V600E [67]. Accordingly, these inhibitors almost eliminated the growth of tumors in vivo with two mutations but had little effect on tumors with BRAF V600E alone [67]. TERT promoter mutations control the apoptosis of BRAF mutant cancer cells, thereby controlling the therapeutic response to BRAF/MEK inhibitors. Therefore, the genetic duet of BRAF V600E and TERT promoter mutations represents an effective therapeutic target in cancer, so that combined targeted therapy may improve the overall survival of melanoma patients with TERT promoter mutations.

#### 4.2.5. Immunotherapy

The development of immunotherapy targeting telomerase is due to its identification as a widely expressed tumor-associated antigen [68]. The endogenous TERT peptides produced by cancer cells can be recognized by major histocompatibility complex (MHC) class I or class II molecules and trigger an adaptive immune response. Telomerase-directed immunotherapy includes vaccines, adoptive cell transfer, and oncolytic virus therapy. Besides, TERT mutations positively correlated with a higher tumor mutational burden (TMB) value, neoantigen load, and tumor purity [43].

In the few studies with evaluable data, in terms of overall survival, immune response to the TERT vaccine is usually found to be associated with clinical benefit. In fact, the overall survival of responders was usually significantly longer, approaching or exceeding twice that of nonresponders. However, data from clinical trials conducted to date indicate that TERT-based therapeutic vaccination has limited anticancer effects: various immunogens have been reported to induce T-cell responses to TERT in cancer patients, but this effect is usually insufficient to control the growth of the tumor. Results of multiple phases I/I-II studies have shown that vaccination based on therapeutic TERT can cause specific T-cell responses in many vaccinators. It has minimal effect on tumor size, and temporary disease stability usually is the best clinical result. In contrast, these clinical studies confirm that the risk of adverse events following vaccination targeting TERT is small or nonexistent [68]. Thus, the TERT vaccine is still worthy of more clinical trials in the future.

Besides, the TERT vaccine has more clinical trials under development, including phase I, II, and III trials. TERT peptide vaccine UV1 elicited an immune response in 86% of patients with metastatic hormone-naive prostate cancer enrolled in phase I/IIa trial [69]. Four vaccines have entered phase II trials, and one of the TERT vaccines (GV1001) has entered phase III. The phase III trial of GV1001 in patients with advanced pancreatic cancer failed to show a survival advantage over chemotherapy [70]. The TERT vaccine has been evaluated in preclinical studies in conjunction with immune checkpoint blockade. The synthetic TERT DNA vaccine works synergistically with anti-CTLA-4 therapy to inhibit tumor growth and prolong survival in mouse models that have a weak response to a single immune checkpoint inhibitor (ICI) [71]. In the subgroup analysis of monotherapy and ICI combination therapy, only in the anticytotoxic T lymphocyte-associated antigen 4 (anti-CTLA4) group did patients with TERT mutations have a better prognosis, especially for melanoma. Therefore, TERT mutations are closely related to higher TMB value and a unique tumor microenvironment, which may be the reason why TERT mutation becomes a potential biomarker for anti-CTLA4 therapy [43]. Further clinical trials are needed to confirm that TERT mutations are potential predictors of anti-CTLA4 therapy, and targeted TERT in combination with immunotherapy may offer better benefits for patients with TERT mutations [43].

Thus, TERT-based immunotherapy may provide opportunities for personalized intervention, may determine the priority of patient selection based on TERT promoter mutations and genomic rearrangement near TERT (molecular profiling), and may be combined with immune checkpoint inhibitors. This approach has the potential to raise cancer immunotherapy to a new level of success [44].

## 5. Conclusion

All in all, the pathogenesis and development of malignant melanoma are complicated and multifactorial. The treatments of malignant melanoma have been constantly making breakthroughs, especially targeted therapy and immunotherapy, in recent years. The above-mentioned exploration of the mechanism of TERT promoter mutations and telomerase in melanoma and related treatment methods are only a small part of the research on malignant melanoma.

TERT promoter mutation is a multiple event and is the most common noncoding mutation in melanoma. Cells with TERT promoter mutations can establish ETS transcription factor binding sites in the promoter to increase TERT promoter transcription levels, upregulate TERT expression in the human body, and increase telomerase activity. It can extend the shortening of telomeres during DNA replication and maintain the length of telomeres. This gives cancer cells unlimited proliferation potential and promotes cell immortalization and melanoma progression. Some studies have shown that TERT promoter mutations can promote the occurrence and development of melanoma, invasion, and metastasis of melanoma and are related to the poor prognosis and survival of melanoma patients. Besides, −138/−139 CC>TT tandem mutations are associated with the worst disease-free survival and specific survival of melanoma. Furthermore, genetic testing and next-generation sequencing can diagnose melanoma patients with TERT promoter mutations. According to different mutation types, their prognosis and survival may be predicted, and targeted treatment options can be selected for mutations. Therefore, it is important to reexamine the therapeutic potential of chemical inhibition of telomerase activity in cancer cells with TERT promoter mutations, as well as some new emerging therapies, such as gene editing, targeted transcription factors, and telomerase inhibition and TERT vaccines. They are worth investigating more in future research, which are promising treatment options to prolong progression-free survival and overall survival of melanoma patients with TERT promoter mutations. Despite the major challenges, TERT promoter mutations and telomerase remain attractive targets for cancer treatment (see Table 1).

## Figures and Tables

**Table 1:** The future therapeutic potential of TERT promoter mutations and telomerase.

Category	Clinical application	Molecular mechanism	References
Gliomas	Gene editing	This validates the feasibility of gene editing as a cancer treatment, and the activated TERT promoter mutation is a cancer-specific therapeutic target	[59]
Glioblastoma	Targeting transcription factor	Reducing TERT expression by disrupting ETS factor GABP*β*1L culminates in telomere loss and cell death exclusively in TERT promoter mutant cells	[62]
Non-small-cell lung cancer	Targeting telomerase	The exploratory analysis demonstrated a trend toward longer median PFS and overall survival in imetelstat-treated patients with short TL, but no improvement in median PFS and OS in patients with long TL	[64]
	Targeting telomerase	Treatment of cancer cells with BIBR1532 leads to progressive telomere shortening, cell proliferation arrest after several weeks of drug treatment, and senescence	[65]
Melanoma	Combined targeted therapy	In melanoma cell lines, after short-term exposure to MAPK inhibitors, regardless of the TERT promoter mutation, a sharp decline in TERT transcription and telomerase activity was observed	[56]
Melanoma	Combined targeted therapy	TERT promoter mutations control the apoptosis of BRAF mutant cancer cells, thereby controlling the therapeutic response to BRAF/MEK inhibitors	[67]
Melanoma	Immunotherapy	In the subgroup analysis of monotherapy and combination ICI treatment, only in the anticytotoxic-T-lymphocyte-associated antigen 4 (anti-CTLA4) group did patients with TERT mutations have a better prognosis, especially for melanoma	[43]
Prostate cancer	Immunotherapy	Treatment with UV1 and GM-CSF gave few adverse events and induced specific immune responses in many unselected patients for HLA type. The intermediate dose of 0.3 mg UV1 resulted in the highest proportion of and most rapid UV1-specific immune responses with an acceptable safety profile	[69]
Pancreatic cancer	Immunotherapy	Adding GV1001 vaccination to chemotherapy did not improve overall survival. New strategies to enhance the immune response effect of telomerase vaccination during chemotherapy are required for clinical efficacy	[70]
	Immunotherapy	We observed that blockade of CTLA-4 or, to a lesser extent, PD-1 synergized with the TERT vaccine, generating more robust antitumor activity compared to checkpoint alone or vaccine alone	[71]

## Data Availability

The data used to support the findings of this study are included within the review.
